# Thermostable Oxidoreductases CotA and Prx Enable Synergistic and Peroxide-Enhanced Degradation of Aflatoxin B_1_

**DOI:** 10.3390/toxins18050193

**Published:** 2026-04-22

**Authors:** Xinyue Zhang, Yufan Yang, Yongping Jiang, Lingfang Shi, Haolan Du, Antonio Francesco Logrieco, Antonio Moretti, Susu Han, Fuguo Xing

**Affiliations:** 1 Key Laboratory of Agro-Products Quality and Safety Control in Storage and Transport Process, Ministry of Agriculture and Rural Affairs, Institute of Food Science and Technology, Chinese Academy of Agricultural Sciences, Beijing 100193, China; zxyyue1213@163.com (X.Z.); dddkayyf@163.com (Y.Y.);; 2 Xianghu Laboratory, Zhejiang Provincial Laboratory of Agriculture, Hangzhou 311231, China; 3 Institute of Sciences of Food Production, Research National Council of Italy, 70126 Bari, Italy

**Keywords:** Aflatoxin B_1_, oxidoreductases, enzymatic degradation, thermostable enzymes, hydrogen peroxide

## Abstract

Aflatoxin B_1_ (AFB_1_) is a highly stable mycotoxin that can persist during conventional food processing and therefore poses a serious risk to food and feed safety. In this study, two enzymes (CotA and Prx) were heterologously expressed in *Bacillus subtilis*, purified by Ni–NTA affinity chromatography, and evaluated for their ability to degrade AFB_1_. Both enzymes exhibited remarkable thermostability and distinct catalytic optima. CotA exhibited its highest activity at 80 °C with an AFB_1_ removal of 38.4%, whereas Prx showed its highest activity at 90 °C with a removal of 82.6%. The optimal pH values were near neutral, with CotA performing best at pH 7.0 and Prx at pH 7.5, and both reactions approached maximal conversion within approximately 10 h. When the two enzymes were combined, a clear cooperative effect was observed. The mixed system achieved 91.0% AFB_1_ removal at 80 °C after 10 h, with the best degradation activity occurring at a CotA to Prx ratio of 1:3. At 50 °C, neither enzyme alone caused appreciable AFB_1_ degradation, but the addition of hydrogen peroxide markedly enhanced catalytic activity. Both enzymes also retained substantial activity after boiling and autoclaving. In a maize flour model, the mixed-enzyme system showed strong AFB_1_ degradation capacity, and peroxide-assisted treatment further improved activity. These results establish a thermostable and peroxide-responsive enzymatic platform for AFB_1_ degradation and support future development of enzyme-based detoxification strategies for food and feed applications. Product identification and toxicological validation will be needed to confirm the safety of the treated products.

## 1. Introduction

AFB_1_, a secondary metabolite mainly produced by *Aspergillus* species such as *A. flavus* and *A. parasiticus*, is classified as a Group 1 carcinogen by the International Agency for Research on Cancer (IARC) because of its potent carcinogenic, mutagenic, and teratogenic effects [1]. It widely contaminates major crops such as maize, peanuts, and wheat, as well as feed raw materials, causing substantial economic losses and posing a serious threat to food safety and public health [2,3]. Owing to the highly stable difuran ring and coumarin lactone ring structures of AFB_1_, conventional thermal processing is often insufficient for its elimination [4]. Therefore, the development of efficient, safe, and environmentally friendly detoxification strategies has become an urgent priority in the food and agricultural sectors [5,6]. At present, AFB_1_ control mainly relies on physical adsorption and chemical degradation. However, these approaches often lead to nutrient loss, damage to food quality, harmful residues, or secondary pollution [7,8,9,10]. In this context, biological detoxification has attracted increasing attention, and enzymatic degradation is considered one of the most promising alternatives because of its mild reaction conditions, substrate specificity, and environmental compatibility [11,12,13].

The enzymatic degradation of AFB_1_ primarily targets its toxicity-determining structural moieties, most notably the C8–C9 unsaturated double bond in the terminal furan ring and the lactone ring. Reported enzymatic detoxification routes involve multiple enzyme classes, including laccases, peroxidases, and certain hydrolases. Among these, laccases play an important role in AFB_1_ degradation. They typically operate via a single-electron oxidation mechanism, generating radical intermediates of AFB_1_ that preferentially attack the C8–C9 double bond of the furan ring, and subsequently trigger bond cleavage, decarboxylation, and lactone ring opening, leading to structural rearrangements. A mechanistic study demonstrated by UPLC–MS that treatment with a recombinant laccase from *Trametes* sp. C30 disrupted both the difuran and lactone ring structures of AFB_1_, yielding multiple transformation products with markedly reduced or negligible toxicity [14].

In contrast, peroxidases are generally considered to reduce AFB_1_ toxicity through peroxide-dependent oxidative modification of key structural sites. These enzymes can catalyze epoxidation of the C8–C9 double bond, forming AFB_1_-8,9-epoxide, which is subsequently hydrolyzed to the 8,9-dihydrodiol derivative [15]. This product exhibits a greatly diminished capacity to form DNA adducts, thereby significantly lowering mutagenic activity and achieving effective detoxification. To date, numerous laccases and peroxidases from diverse biological sources have been reported to enhance AFB_1_ degradation efficiency. Collectively, although laccases and peroxidases differ in catalytic mechanisms, previous studies have shown that enzymatic oxidation may target the C8–C9 double bond and the lactone-associated structure of AFB_1_. However, the transformation products generated by the present CotA–Prx system remain to be identified.

However, due to the potential lytic effects of laccases on host cell walls and the oxidative stress imposed by peroxidases on host cells, the production levels of these enzymes in their native hosts are often limited. Enzyme production is strongly influenced by strain background and cultivation conditions, yet under conventional liquid fermentation the overall secretion levels remain relatively low. In non-engineered natural hosts without genetic modification or fermentation optimization, the activities of laccases and peroxidases are typically in the range of single digits to several tens of U/mL. For example, laccase activities produced by different white-rot fungi generally range from 0.5 to 75 U/mL [16]. Such levels are insufficient to meet the demands of large-scale industrial applications requiring high enzymatic activity and stability. Therefore, the establishment of efficient heterologous expression systems has become a key strategy to enhance production and enable practical application. *B. subtilis* is an attractive cell factory due to its high protein secretion capacity, robustness under fermentation conditions, and suitability for extracellular enzyme production. Notably, CotA laccase achieved efficient secretory expression in *B. subtilis* WB600 through signal-peptide screening [17]. More recently, heterologous secretory expression of several small bacterial laccases has also been demonstrated in *B. subtilis*, further supporting its capacity as a secretion-oriented production host [18]. However, AFB_1_-centered studies that integrate both aspects into a unified, mechanism-informed detoxification framework remain relatively limited. Thus, heterologous expression in *B. subtilis* represents a promising strategy to improve the production of laccases and peroxidases. In addition, CotA has been previously implicated in AFB_1_ oxidation, while Prx-family enzymes are associated with peroxide-dependent redox transformations and may influence oxidative conversion routes [19,20,21]. Consequently, the in vitro synergistic enzymatic degradation systems composed of different laccases and peroxidases could provide complementary catalytic functions, broaden the effective operational window, and improve detoxification performance under practically relevant conditions [22,23].

In this study, two *Bacillus*-derived oxidoreductases, CotA and Prx, were heterologously expressed in *B. subtilis* and evaluated for their capacity to degrade AFB_1_. We characterized the effects of temperature, pH, and metal ions on their catalytic ability, examined the ability of synergistic degradation for AFB_1_, and assessed the peroxide responsiveness of the system at a moderate temperature. In addition, we investigated the thermal processing tolerance of both enzymes and evaluated their applicability in a maize flour model. This work aimed to establish a thermostable enzymatic platform for AFB_1_ reduction and to provide a basis for future mechanistic and application-oriented studies.

## 2. Results

### 2.1. Heterologous Expression and Purification of CotA and Prx in B. subtilis

CotA and Prx were successfully expressed in *B. subtilis* as soluble proteins. SDS–PAGE analysis showed prominent bands at the expected molecular weights of approximately 65 kDa for CotA and 25 kDa for Prx (Figure S1).

### 2.2. Both Enzymes Catalyze AFB_1_ Removal with Distinct Temperature Optima and Extreme Thermotolerance

**Figure 1:**
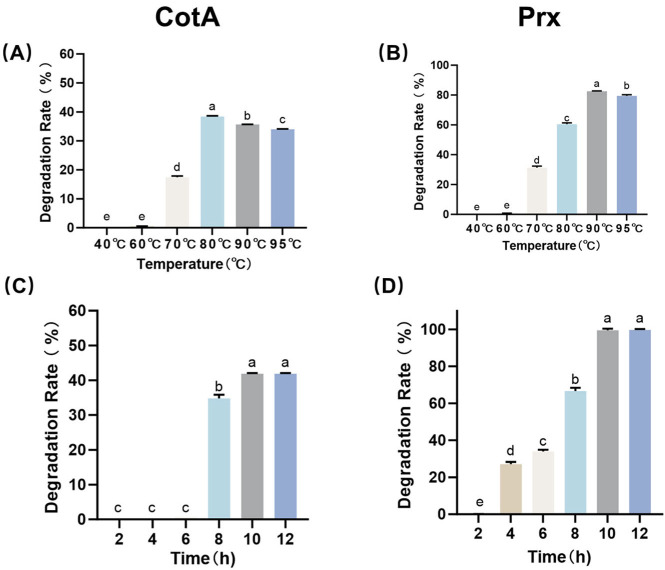
Temperature and time dependence of AFB_1_ degradation catalyzed by CotA and Prx. (**A**) Effect of temperature on CotA activity. (**B**) Effect of temperature on Prx activity. (**C**) Time-course degradation of AFB_1_ by CotA at its optimal temperature. (**D**) Time-course degradation of AFB_1_ by Prx at its optimal temperature. Values represent mean ± SD (*n* = 3). Different letters indicate significant differences (*p* < 0.05).

Temperature profiling demonstrated that CotA showed the highest AFB_1_ degradation rate at 80 °C, achieving a degradation rate of 38.4% under standard assay conditions (Figure 1A). In contrast, Prx displayed its highest activity at 90 °C with an AFB_1_ degradation rate of 82.6% (Figure 1B). Across the tested range, both enzymes retained measurable detoxification capacity at elevated temperatures, indicating an unusually high thermal tolerance consistent with their origin as thermostable *Bacillus* enzymes. Time-course experiments further showed that the AFB_1_ degradation rate increased with incubation time and approached a maximum after 10 h for CotA and 10 h for Prx at their respective optimal temperatures (Figure 1C,D and Figure S2).

### 2.3. pH Dependence of AFB_1_ Degradation

**Figure 2:**
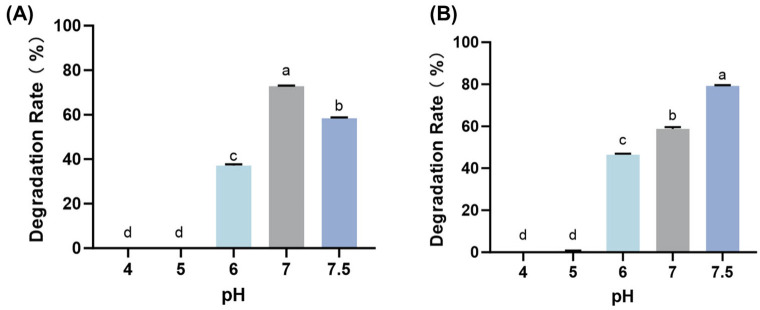
pH dependence of AFB_1_ degradation catalyzed by CotA (**A**) and Prx (**B**). AFB_1_ degradation rates were determined by HPLC analysis under the optimal temperature conditions for each enzyme. Values represent mean ± SD (*n* = 3). Different letters indicate significant differences (*p* < 0.05).

CotA showed the highest AFB_1_ degradation activity at pH 7, with a maximum degradation rate of 72.6% (Figure 2A). Prx showed optimal activity at pH 7.5, reaching a maximum degradation rate of 79.2% (Figure 2B).

### 2.4. Influence of Metal Ions on AFB_1_ Degradation

**Figure 3:**
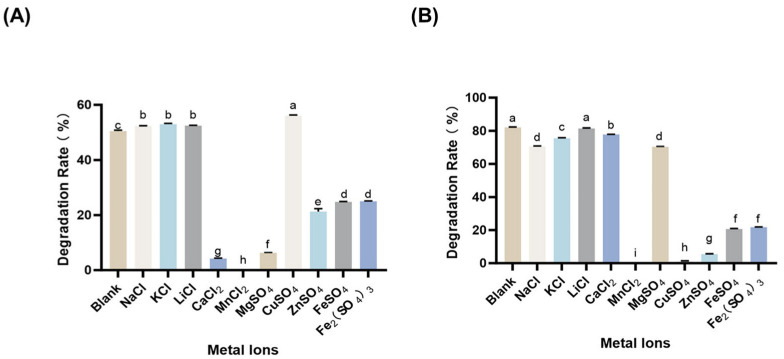
Effect of metal ions on AFB_1_ degradation catalyzed by CotA (**A**) and Prx (**B**). Reactions were conducted in the presence of different metal ions under the respective optimal conditions of each enzyme. AFB_1_ degradation was quantified by HPLC. Values represent mean ± SD (*n* = 3). Different letters indicate significant differences (*p* < 0.05).

The effects of metal ions on AFB_1_ degradation performance were systematically assessed to inform practical formulation and to probe potential catalytic requirements (Figure 3A). CotA-mediated AFB_1_ degradation activity was enhanced in the presence of Cu^2+^ (as well as Na^+^, K^+^, and Li^+^), whereas Mn^2+^ caused near-complete inhibition of CotA activity. In addition, Ca^2+^ and Mg^2+^ strongly inhibited CotA activity, and led to 91.7% and 87.5% activity loss, respectively, at 2 mM.

Prx displayed a distinct ion-response profile. Mn^2+^ also almost completely inhibited Prx activity, and Prx was highly influenced by Cu^2+^ and Zn^2+^, retaining only 2.7% and 6.6% residual activity, respectively, at 2 mM. Collectively, these results indicated that the two enzymes respond differently to ionic environments, which is important when designing combined enzyme formulations and when considering complex food and feed matrices (Figure 3B).

### 2.5. In Vitro Synergistic Enzymatic Degradation of AFB_1_

**Figure 4:**
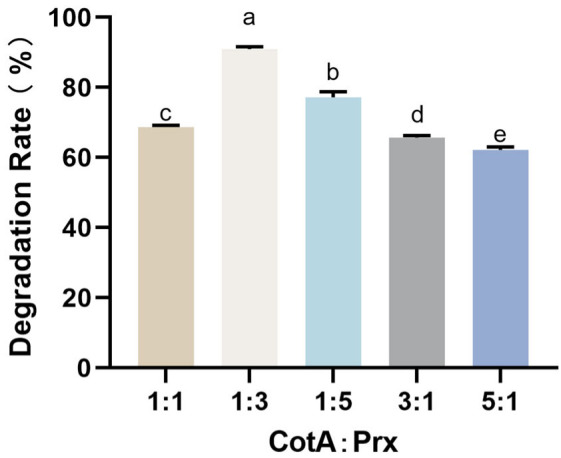
Influence of the CotA–Prx mixing ratio on AFB_1_ degradation. Reactions were performed with different molar ratios of CotA and Prx while maintaining the same total enzyme concentration. Values represent mean ± SD (*n* = 3). Different letters indicate significant differences (*p* < 0.05).

To examine whether CotA and Prx act synergistically in AFB_1_ degradation, the two enzymes were combined at different molar ratios while keeping the total enzyme concentration constant, and the AFB_1_ removal efficiency was determined. Under 80 °C incubation for 10 h, the mixed-enzyme system achieved 91.0% AFB_1_ removal. The degree of enhancement depended on the mixing ratio, with the strongest performance observed at CotA: Prx = 1:3 (Figure 4). These results indicated a clear ratio-dependent enhancement when CotA and Prx were combined. The best performance at a CotA: Prx molar ratio of 1:3 suggested functional complementarity between the two enzymes; however, the mechanistic basis of this enhancement remains to be clarified by product profiling and sequential reaction analysis.

### 2.6. Hydrogen Peroxide Markedly Boosted AFB_1_ Removal at Moderate Temperature

**Figure 5:**
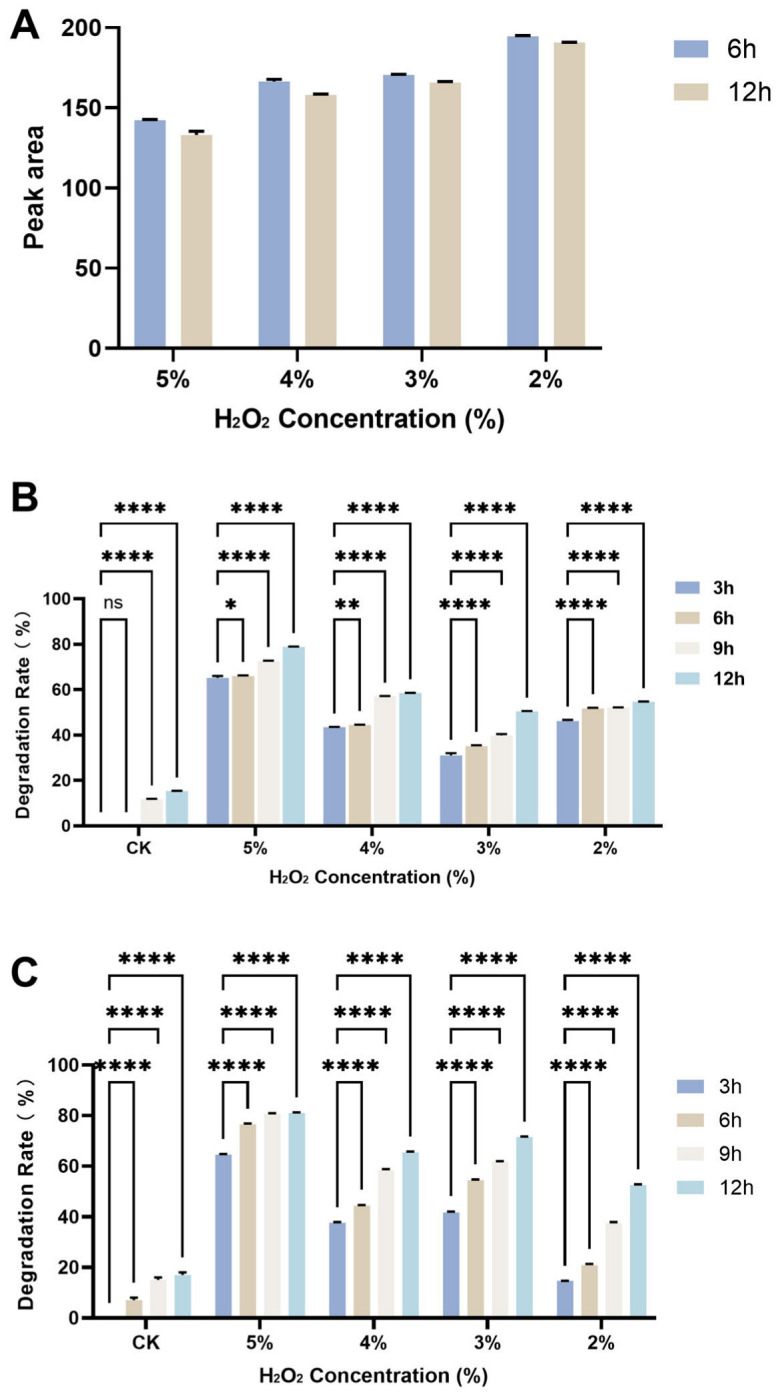
Effect of H_2_O_2_ on AFB_1_ reduction at 50 °C. (**A**) Residual AFB_1_ peak area in the H_2_O_2_-only system. (**B**) Time-dependent AFB_1_ reduction by CotA in the presence of different H_2_O_2_ concentrations. (**C**) Time-dependent AFB_1_ reduction by Prx in the presence of different H_2_O_2_ concentrations. Data are presented as mean ± SD (*n* = 3). Statistical significance is indicated as ns, * *p* < 0.05, ** *p* < 0.01 and **** *p* < 0.0001.

At 50 °C, H_2_O_2_ alone showed no appreciable effect on AFB_1_ degradation. As shown in Figure 5A, HPLC analysis indicated that the AFB_1_ peak area remained essentially unchanged over the incubation period. Likewise, CotA and Prx exhibited negligible AFB_1_ degradation at 50 °C (Figure 1A), indicating that temperature is the key limiting factor for catalytic reduction under these conditions. H_2_O_2_ at concentrations of 2% or lower did not promote enzymatic degradation of AFB_1_. Notably, however, the combinations of CotA + 5% H_2_O_2_ and Prx + 5% H_2_O_2_ achieved substantially higher AFB_1_ removal at 50 °C, reaching 78.9% and 81.0%, respectively (Figure 5B,C). These results suggested that H_2_O_2_ promotes AFB_1_ conversion at moderate temperature by increasing the effective oxidative driving force of the CotA and Prx based systems.

### 2.7. Molecular Docking of AFB_1_ with Enzymes

**Figure 6:**
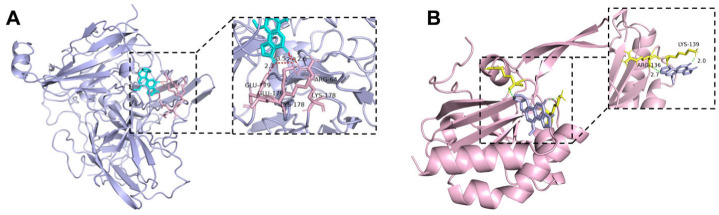
Molecular docking models of AFB_1_ binding to CotA (**A**) and Prx (**B**). Insets show enlarged views of the active-site interactions between AFB_1_ and surrounding residues. Hydrogen bonds and interaction distances are indicated.

Docking outputs for AFB_1_ are provided in Table S1. Under the same docking settings, CotA produced a more favorable binding score (−7.86 kcal/mol) than Prx, and the top-ranked CotA pose displayed a greater number of polar contacts. Specifically, the best CotA pose contained four hydrogen bonds between AFB_1_ and pocket residues (Figure 6A), whereas the best Prx pose involved two hydrogen bonds (Figure 6B). Overall, the higher contact density in CotA was consistent with tighter anchoring of AFB_1_ in the pocket in silico.

### 2.8. Detoxification Capacity Persists After Boiling and Autoclaving

**Figure 7:**
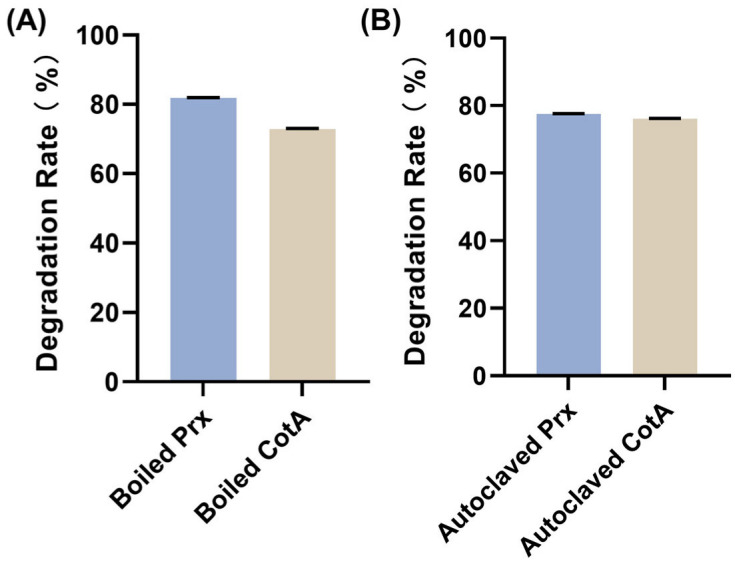
Residual AFB1 degradation activity of CotA and Prx after thermal treatments. Enzymes were subjected to (**A**) boiling (100 °C, 15 min) and (**B**) autoclaving (121 °C, 15 min) prior to the degradation assay. AFB1 degradation was quantified by HPLC. Values represent mean ± SD (*n* = 3).

Given the exceptional thermostability of both enzymes, we investigated whether harsh thermal processing steps used in industrial sterilization would abolish their detoxification capacity. After boiling at 100 °C for 15 min, CotA and Prx retained substantial AFB_1_ reducing activity, with degradation rates of 72.8% and 81.9%, relative to the untreated controls (Figure 7A). Notably, even after autoclaving at 121 °C for 15 min, CotA and Prx still showed degradation rates of 76.1% and 77.4% (Figure 7B).

### 2.9. Application of the Enzyme System in a Maize-Based Matrix

**Figure 8:**
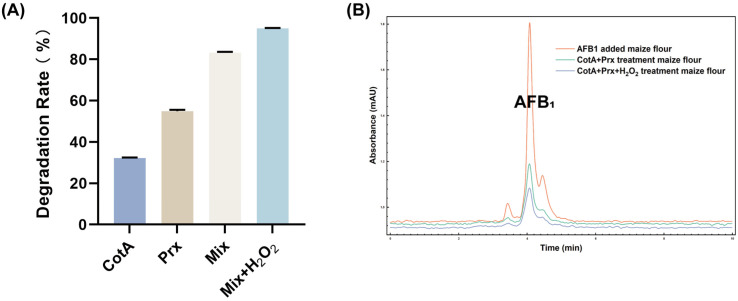
(**A**) Comparison of AFB_1_ degradation efficiency by CotA, Prx, their enzyme mixture, and the peroxide-assisted system. CotA and Prx were tested individually, in combination (Mix), and in the presence of hydrogen peroxide (Mix + H_2_O_2_). (**B**) HPLC chromatograms of the CotA–Prx mixed-enzyme system and the mixed-enzyme plus hydrogen peroxide treatment, compared with the control of AFB_1_-added maize flour. Values represent mean ± SD (*n* = 3).

To assess performance in a realistic food and feed matrix, a maize flour model spiked with AFB_1_ at 50 μg/kg was established. Treatment with CotA or Prx individually resulted in AFB_1_ removal rates ranging from 32% to 55%, whereas the mixed-enzyme system achieved an 83% removal rate under the same conditions. When H_2_O_2_ assistance was introduced at moderate temperature (50 °C), the removal rate increased to 95% without detectable matrix-induced inhibition (Figure 8). These results demonstrated the practical degradation potential of the CotA/Prx system and support subsequent optimization toward scalable processing.

## 3. Discussion

AFB_1_ is unusually difficult to control because its toxicity is tied to chemically stable structural motifs that survive many routine processing steps. This work showed that two enzymes, CotA and Prx, can remove AFB_1_ under conditions that are atypical for most reported AFB_1_ degradation enzymes, particularly at a high temperature. CotA and Prx exhibited distinct optimal temperatures and thermal limits, with CotA peaking at 80 °C and Prx peaking at 90 °C (Figure 1A,B). Under their respective optimal temperatures, both reactions reached maximal conversion after approximately 10 h. In contrast, the fermentation broth of *B. subtilis* HNGD-Mq02 operated under much milder conditions, showing maximal activity at 50 °C and an optimal pH of 7.0 [24]. Its time-course profile indicated rapid conversion during the first 48 h, followed by a plateau with little further improvement after 72 h (Figure 1C,D). This comparison suggested that the CotA/Prx system in the present study achieved a shorter reaction time at the expense of a higher operating temperature, whereas the HNGD-Mq02 broth system favored moderate temperature but required longer incubation to reach equilibrium. A similar low-to-moderate temperature with longer duration pattern has also been observed for recombinant fungal laccases such as rAnLI, which achieved high AFB_1_ degradation efficiency at 35 °C and pH 5.0 with a reaction time of 48 h [25]. Moreover, not all laccases can directly oxidize AFB_1_ without auxiliary chemistry. Ery4 laccase was reported to be unable to directly oxidize AFB_1_, and AFB_1_ removal was only achieved after introducing redox mediators under 25 °C for 72 h, highlighting that AFB_1_ detoxification by oxidoreductases is highly dependent on the specific enzyme and reaction system [26].

Both enzymes showed near-neutral pH optima, with CotA performing best at pH 7.0 and Prx at pH 7.5 (Figure 2), which is advantageous for practical handling and can help minimize confounding chemical conversion. This is important because AFB_1_ can become unstable under extreme acidity or alkalinity, where lactone-ring hydrolysis and ring opening may occur and lead to apparent loss that is not enzyme-driven. Therefore, conducting assays near neutral pH strengthens the interpretation that the observed AFB_1_ decrease primarily reflects enzymatic transformation rather than pH-induced breakdown [27]. It is noteworthy that AFB_1_ itself is unstable under strongly acidic (pH ≤ 3) and strongly alkaline (pH ≥ 8) conditions. Under extreme pH environments, the lactone ring of AFB_1_ is prone to ring opening, leading to structural disruption and non-enzymatic degradation. Therefore, when evaluating enzymatic detoxification efficiency, the intrinsic chemical stability of AFB_1_ across different pH conditions should be carefully considered [4].

Metal ions exhibited pronounced but non-uniform effects on CotA- and Prx-mediated detoxification, and the ion-response pattern differed from that reported for the BsCotA–methyl syringate system. In the present results, CotA activity was enhanced by Cu^2+^ and by monovalent cations Na^+^, K^+^, and Li^+^, whereas Mn^2+^ almost eliminated CotA activity and Ca^2+^ and Mg^2+^ caused strong losses at 2 mM (Figure 3). Prx showed a distinct sensitivity profile, with Mn^2+^ again nearly abolishing activity and Cu^2+^ and Zn^2+^ leaving only minimal residual activity at 2 mM, indicating that ion environments could differentially inhibit the two components in a mixed-enzyme formulation. By contrast, in the BsCotA–methyl syringate system, K^+^, Ca^2+^, Na^+^, Mg^2+^, and Li^+^ were reported to have no obvious effect on AFB_1_ and ZEN removal under conditions where the toxins were nearly completely degraded after 10 h, while Cu^2+^ and Mn^2+^ reduced AFB_1_ degradation to 74.0% and 86.6%, respectively, and strong inhibition was observed with Cr^3+^ and Al^3+^ (Figure 3). This comparison suggested that ion effects are highly system-dependent and can change in both magnitude and direction with enzyme origin and reaction configuration, including whether detoxification is driven by direct enzyme action or by a laccase–mediator process [28].

CotA and Prx displayed non-additive behavior when combined, reaching 91.0% AFB_1_ removal at 80 °C for 10 h and outperforming either enzyme alone. The dependence on mixing ratio, with the best performance at CotA to Prx of 1:3 (Figure 4), supported functional complementarity rather than a simple increase in total enzyme mass. Similar behavior has been reported for dual-enzyme AFB_1_ detoxification systems. For example, a combination of two peroxidases, Il-MnP1 and Il-DyP4, achieved higher AFB_1_ degradation than either enzyme alone and was associated with a broader product spectrum, consistent with the idea that different enzymes can act on different reactive sites and collectively increase conversion flux [29]. In addition, coupled enzyme designs have been used to strengthen oxidative driving force for AFB_1_ conversion, such as manganese peroxidase systems supported by an auxiliary enzyme that supplies peroxide, highlighting that multi-enzyme cooperation can arise from either sequential substrate transformation or complementary redox roles. Nevertheless, as in prior dual-enzyme studies where product analysis was essential to substantiate pathway claims, definitive coupling between CotA and Prx mediated steps still requires product profiling under single-enzyme and mixed-enzyme conditions [30].

At 50 °C, neither H_2_O_2_ alone nor enzyme alone produced appreciable AFB_1_ removal (Figure 5), indicating that moderate temperature is a key limiting factor for catalysis in this system. The marked enhancement observed after adding 5% H_2_O_2_ to either CotA or Prx supported the role of peroxide as an external oxidative driving force that can enable or accelerate AFB_1_ conversion when thermal activation is insufficient. This interpretation is consistent with peroxide-dependent AFB_1_ transformation systems reported for peroxidase-type oxidoreductases, where H_2_O_2_ is supplied as a required co-substrate to initiate catalytically competent oxidative intermediates, as exemplified by DyP-mediated degradation assays that explicitly include H_2_O_2_ in the reaction mixture [31]. Moreover, the need to control oxidant dosage was supported by MnP-based detoxification studies showing that moderate H_2_O_2_ levels can enhance activity whereas higher concentrations can inhibit the enzyme, highlighting a practical trade-off between reaction driving force and oxidative deactivation [32]. Together, these comparisons support discussing peroxide addition as a controllable strategy to expand the operational window of CotA- and Prx-based degradation toward moderate temperature, while emphasizing that product profiling and oxidant management are necessary to avoid non-productive side reactions under peroxide-enhanced conditions. A practical limitation of the present system is that the intrinsic activity of CotA and Prx was strongest at relatively high temperatures. Although H_2_O_2_ broadened the operating window and enabled substantial AFB_1_ conversion at 50 °C, the peroxide concentration used here mainly served to demonstrate the biochemical responsiveness of the system. Further optimization will be required to reduce oxidant dosage and to balance toxin conversion with preservation of the food and feed matrix.

Docking results provided a plausible structural basis for the different behaviors of CotA and Prx toward AFB_1_. CotA formed a more favorable docking score than Prx and formed more hydrogen bonds in the top-ranked pose (Figure 6), which was consistent with tighter anchoring of AFB_1_ in the CotA pocket in silico. While docking does not prove catalysis, these differences supported a model in which CotA provided a more stable binding environment for AFB_1_, which may help explain why higher degradation activity was still observed above 80 °C.

The retention of high degradation capacity after boiling and after autoclaving is notable and practically relevant. Residual AFB_1_ removal remained high for both enzymes after boiling for 15 min and after 121 °C sterilization for 15 min (Figure 7), indicating that the functional structures are resilient to severe thermal handling. This property reduced barriers to deployment because enzymes can be sterilized and stored with simpler protocols, which are especially useful for food or feed processing environments where microbial control is required.

Performance in the maize flour matrix further supported application potential and highlighted the importance of system design. Single enzymes achieved moderate removal, whereas the mixed system reached 83% under the same matrix conditions, and peroxide assistance at moderate temperature increased removal to 95% (Figure 8). Matrix effects commonly reduce in vitro efficacy in cereal systems because toxin accessibility and competing adsorption sites limit enzyme–substrate contact. A related corn-flour study reported that AFB_1_ reduction in corn was substantially lower than in vitro and emphasized the need to evaluate matrix effects and oxidative impacts alongside toxin reduction. In this context, the higher removal achieved here suggests improved matrix compatibility, while the peroxide-assisted approach still warrants attention to oxidative side effects in the matrix during optimization.

Although a substantial decrease in the AFB_1_ peak area was observed in both the in vitro system and the maize flour model, the transformation products were not identified in the present study. Therefore, the current data demonstrated efficient reduction in the parent AFB_1_ signal, but not yet the detailed conversion pathway. LC–MS/MS-based product identification will be necessary in future work to determine whether key toxicity-associated structural moieties, such as the C8–C9 double bond or lactone-related structure, are disrupted by the CotA–Prx system. At the same time, future studies should further examine the mutagenicity of the CotA–Prx degradation system in order to verify the safety of the resulting products.

## 4. Conclusions

In summary, this study established a thermostable CotA–Prx enzymatic platform for AFB_1_ reduction. The two enzymes exhibited distinct high-temperature catalytic optima and showed clear ratio-dependent enhancement when combined, with the best performance at a CotA:Prx molar ratio of 1:3. H_2_O_2_ further expanded the operating window of the system by enabling substantial AFB_1_ conversion at 50 °C, and the enzyme platform remained functional after severe thermal treatment and in a maize flour matrix. However, identification of transformation products and toxicological validation of the treated samples are still required before practical food and feed application can be fully justified.

## 5. Materials and Methods

### 5.1. Chemicals and Reagents

AFB_1_ standard (≥98%) was purchased from Pribolab (Qingdao, China) and prepared as a stock solution in HPLC-grade methanol at 1 mg/mL, stored at −20 °C. Hydrogen peroxide (H_2_O_2_, 30%, *w*/*w*) and all analytical-grade salts and buffer components were obtained from Colaber (Beijing, China). Ultrapure water (18.2 MΩ·cm) was used throughout. Unless otherwise stated, all degradation assays were conducted in the dark to minimize photodegradation.

### 5.2. Strains, Plasmids, and Gene Construction

The coding sequences of cotA and prx were amplified from genomic DNA of *B. velezensis* B.26. The target genes were cloned into the *B. subtilis* expression vector pMA5 using a prolonged overlap extension PCR (POE-PCR) strategy. Primers were designed with 20–25 bp homologous overlaps to the vector backbone to enable seamless assembly [33].

PCR amplification was carried out using Phusion high-fidelity DNA polymerase (Thermo, Waltham, MA, USA) under the following conditions: initial denaturation at 98 °C for 30 s; 30 cycles of 98 °C for 10 s, 60 °C for 10 s, and extension at 72 °C (3 kb min^−1^). PCR products were purified and mixed with the linearized vector at equimolar ratios, followed by overlap extension PCR without primers to generate multimeric plasmids. The extension time was set to 1.5–2 times longer than standard conditions to promote multimer formation. The resulting DNA products were directly used for transformation. For intracellular expression and purification, an N-terminal His_6_-tag was introduced. All constructs were verified by Sanger sequencing (Sangon Biotech, Shanghai, China).

Super-competent cells were prepared using *B. subtilis* SCK6. A single colony was cultured in LB medium containing erythromycin (0.3 μg mL^−1^) at 37 °C and 200 rpm for 8–12 h. The culture was diluted to OD_600_ ≈ 1.0, and D-xylose (1%, *w*/*v*) was added to induce ComK overexpression. After 2 h incubation, cells were used immediately for transformation.

For transformation, 1–2 μL of multimeric plasmid DNA was added to 100 μL of freshly prepared competent cells and incubated at 37 °C for 1.5 h with shaking. Cells were plated on LB agar supplemented with the appropriate antibiotic and incubated at 37 °C for 8–12 h. Positive colonies were screened by colony PCR and confirmed by sequencing.

Recombinant *B. subtilis* strains harboring cotA or prx were cultivated in LB medium with antibiotic at 37 °C. When OD_600_ reached 0.6–1.0, expression was induced with 1% (*w*/*v*) D-xylose and continued for 4–12 h [34]. Cells were harvested and disrupted by sonication at 4 °C. His_6_-tagged proteins were purified by Ni-NTA affinity chromatography [35]. Protein purity was analyzed by SDS-PAGE, and enzymatic activity was determined using substrate-specific spectrophotometric assays.

### 5.3. Heterologous Expression in B. subtilis

Recombinant *B. subtilis* strains were cultivated in TB medium at 37 °C with shaking at 220 rpm. Cells were harvested after 48 h. For intracellular proteins, pellets were washed and resuspended in lysis buffer (50 mM Tris-HCl, 300 mM NaCl, pH 8.0). Cell disruption was performed by sonication on ice. For secreted proteins, culture supernatants were collected by centrifugation and filtered (0.22 µm) prior to purification [36,37].

### 5.4. Protein Purification and Characterization

CotA and Prx were purified using Ni^2+^-NTA affinity chromatography (Genescript, Nanjing, China). Briefly, the crude enzymes were loaded onto a pre-equilibrated Ni^2+^-NTA column with buffer A (50 mM Tris-HCl, 300 mM NaCl, 20 mM imidazole, pH 8.0), and the unbound proteins were washed with 15 column volumes (CV) of buffer A. The bound proteins were eluted with buffer B (50 mM Tris-HCl, 300 mM NaCl, 250 mM imidazole, pH 8.0). Protein purity was assessed by SDS–PAGE, and concentration was determined using the Bradford or BCA method with bovine serum albumin as a standard [38,39,40].

### 5.5. HPLC Quantification of Residual AFB_1_ and Calculation of Degradation Rate

AFB_1_ was quantified by HPLC using an Agilent 1260 instrument (Santa Clara, CA, USA) equipped with a fluorescence detector. Separation was performed on a C18 column (4.6 mm × 250 mm, 5 μm) at 30 °C. The mobile phase consisted of methanol and water, using an isocratic elution of 70:30 (*v*/*v*). The flow rate was set at 1.0 mL/min, and the injection volume was 20 μL. AFB_1_ was detected at fluorescence excitation and emission wavelengths of 365 and 435 nm, respectively. Under these conditions, the retention time of AFB_1_ was approximately 4.2 min [41].

### 5.6. Temperature and pH Profiling

To determine temperature optima, reactions were incubated at temperatures ranging from 20 to 95 °C at a fixed pH 7 for 10 h. For pH optima, a buffer set covering pH 3–10 was used (e.g., citrate–phosphate for pH 3.0–6.0, phosphate for pH 6.0–8.0, Tris-HCl for pH 8.0–9.0, glycine–NaOH for pH 9.0–11.0), while temperature was fixed at each enzyme’s optimal temperature. The optimal temperature and pH were defined as conditions yielding maximal AFB_1_ removal [42,43].

### 5.7. Effect of Metal Ions on AFB_1_ Degradation

The effects of metal ions and additives on enzymatic degradation of AFB_1_ were investigated by individually adding metal salts or other additives (e.g., Cu^2+^, Mn^2+^, Fe^2+^/Fe^3+^, Zn^2+^, Mg^2+^, Ca^2+^, Co^2+^, and Ni^2+^) to the reaction mixtures at a final concentration of 2 mM. The mixtures were incubated for 12 h under the optimal reaction conditions determined for each enzyme [44,45]. Residual AFB_1_ was then quantified by HPLC as described in Section 5.5, and the degradation rate was calculated accordingly.

### 5.8. Mixed-Enzyme Assay and Synergy Analysis

CotA and Prx were combined at defined molar ratios (1:0, 3:1, 1:1, 1:3, and 0:1) while keeping the total enzyme concentration constant at 0.5 μM. The reaction mixtures were incubated under the selected assay conditions, and residual AFB_1_ was quantified by HPLC.

### 5.9. Hydrogen Peroxide-Assisted Degradation

To evaluate the effect of H_2_O_2_ at a moderate temperature, reactions were conducted at 50 °C with varying H_2_O_2_ concentrations (0.3–5%, *v*/*v*). The following conditions were tested: H_2_O_2_ only, CotA only, Prx only, and enzyme plus H_2_O_2_ [46]. All reactions were incubated at 50 °C for 10 h and quenched by adding an equal volume of methanol. Residual AFB_1_ in the reaction mixtures was quantified by HPLC.

### 5.10. Molecular Docking of AFB_1_ with Enzymes

AFB_1_ was used as the ligand for molecular docking, and its three-dimensional structure was retrieved from the PubChem database. The tertiary structures of CotA and Prx were generated by homology modeling using the SWISS-MODEL server, with CotA (PDB: 2WSD) and Prx (PDB: 7KQ6) selected as templates. Template selection prioritized sequence identity > 20% and structural coverage > 85% to ensure model reliability. The resulting models were inspected in PyMOL 2.5 (Schrödinger, NY, USA) to confirm overall folding and the integrity of the putative catalytic pocket [47,48,49].

Molecular docking was performed using AutoDock Vina (v1.2.0) under a semi-flexible protocol, in which the ligand was treated as flexible and the receptor as rigid. Protein structures were prepared by removing crystallographic water molecules, adding polar hydrogens, assigning Gasteiger charges, and converting to PDBQT format. AFB_1_ was energy-minimized, partial charges were calculated, and rotatable bonds were defined prior to docking. For each enzyme, a docking grid box was centered on the predicted catalytic pocket (including the substrate-entry region when applicable) and set to 126 × 126 × 126 Å to fully cover the active-site cavity. Docking was executed with default grid spacing and exhaustiveness settings, and 10 binding poses were generated for each protein–ligand pair. Poses were ranked by predicted binding affinity, and the top-ranked conformation was selected as the representative binding mode. The binding interactions and ligand orientation were visualized and rendered in PyMOL 2.5 [50].

### 5.11. Thermal Processing Tolerance: Boiling and Autoclaving

Purified enzymes were subjected to (i) boiling (100 °C, 15 min) and (ii) autoclaving (121 °C, 15 min, 0.1 MPa). After cooling to room temperature, treated enzymes were used for AFB_1_ degradation assays at optimal temperature and pH of each enzyme. Residual degradation capacity was expressed as a percentage of untreated enzyme activity under the same assay conditions.

### 5.12. Application in a Maize-Based Model System

To evaluate applicability, corn flour was spiked with AFB_1_ to 50 μg/kg and left overnight to evaporate the spiking solvent. Enzymes were added at 5 U/mL in sodium acetate buffer and incubated at 80 °C for 10 h with shaking. After centrifugation, both supernatant and pellet were analyzed. AFB_1_ was extracted with methanol/water (70:30, *v*/*v*) and quantified by HPLC.

### 5.13. Statistical Analysis

All experiments were performed with at least three independent replicates unless otherwise stated. Data are presented as mean ± SD. Statistical significance was evaluated using one-way ANOVA followed by Tukey’s multiple-comparison test, with *p* < 0.05 considered statistically significant. For column charts, significant differences are indicated by different letters. For the time-course data shown in Figure 5, significance is indicated using the asterisk system (ns, *, **, ***, ****).

## Data Availability

The original contributions presented in this study are included in the article/Supplementary Material. Further inquiries can be directed to the corresponding authors.

## Supplementary Materials

The following supporting information can be downloaded at: https://www.mdpi.com/article/10.3390/toxins18050193/s1, Figure S1: SDS-PAGE analysis of CotA and Prx expressed in *B. subtilis* SCK6; Figure S2: HPLC chromatograms of the time course of AFB_1_ degradation by CotA and Prx; Table S1: Docking score and hydrogen bonding distances between enzymes and AFB_1_.

## References

[1] Chen L., Guo W., Zheng Y., Zhou J., Liu T., Chen W., Liang D., Zhao M., Zhu Y., Wu Q. (2020). Occurrence and Char-acterization of Fungi and Mycotoxins in Contaminated Medicinal Herbs. Toxins.

[2] Wang L., Huang Q., Wu J., Wu W., Jiang J., Yan H., Huang J., Sun Y., Deng Y. (2022). The Metabolism and Biotransformation of AFB1: Key Enzymes and Pathways. Biochem. Pharmacol..

[3] Alim M., Iqbal S.Z., Mehmood Z., Asi M.R., Zikar H., Chanda H., Malik N. (2018). Survey of Mycotoxins in Retail Market Cereals, Derived Products and Evaluation of Their Dietary Intake. Food Control.

[4] Martínez J., Hernández-Rodríguez M., Méndez-Albores A., Téllez-Isaías G., Mera Jiménez E., Nicolás-Vázquez M.I., Miranda Ruvalcaba R. (2023). Computational Studies of Aflatoxin B1 (AFB1): A Review. Toxins.

[5] Vidal A., Mengelers M., Yang S., De Saeger S., De Boevre M. (2018). Mycotoxin Biomarkers of Exposure: A Comprehensive Review. Comp. Rev. Food Sci. Food Safe.

[6] Penagos-Tabares F., Khiaosa-ard R., Nagl V., Faas J., Jenkins T., Sulyok M., Zebeli Q. (2021). Mycotoxins, Phytoestrogens and Other Secondary Metabolites in Austrian Pastures: Occurrences, Contamination Levels and Implications of Geo-Climatic Factors. Toxins.

[7] Hojnik N., Modic M., Walsh J.L., Žigon D., Javornik U., Plavec J., Žegura B., Filipič M., Cvelbar U. (2021). Unravelling the Pathways of Air Plasma Induced Aflatoxin B1 Degradation and Detoxification. J. Hazard. Mater..

[8] Tapingkae W., Srinual O., Lumsangkul C., Doan H.V., Chiang H.-I., Manowattana A., Boonchuay P., Chaiyaso T. (2022). In-dustrial-Scale Production of Mycotoxin Binder from the Red Yeast Sporidiobolus Pararoseus KM281507. J. Fungi.

[9] Zhu Y., Hassan Y., Lepp D., Shao S., Zhou T. (2017). Strategies and Methodologies for Developing Microbial Detoxification Systems to Mitigate Mycotoxins. Toxins.

[10] Arimboor R. (2024). Metabolites and Degradation Pathways of Microbial Detoxification of Aflatoxins: A Review. Mycotoxin Res..

[11] Liu M., Zhang X., Luan H., Zhang Y., Xu W., Feng W., Song P. (2024). Bioenzymatic Detoxification of Mycotoxins. Front. Microbiol..

[12] Guo Y., Qin X., Tang Y., Ma Q., Zhang J., Zhao L. (2020). CotA Laccase, a Novel Aflatoxin Oxidase from Bacillus licheniformis, Transforms Aflatoxin B1 to Aflatoxin Q1 and Epi-Aflatoxin Q1. Food Chem..

[13] Zhang A., Yang J. (2025). A Review of Research Progress on the Microbial or Enzymatic Degradation and Mechanism of Aflatoxin B1. J. Microbiol. Biotechnol..

[14] Liu Y., Mao H., Woldemariam Yohannes K., Wan Z., Cao Y., Tron T., Lin J., Jiang Y., Li H., Wang J. (2021). Degradation of Aflatoxin B1 by a Recombinant Laccase from Trametes sp. C30 Expressed in Saccharomyces Cerevisiae: A Mechanism As-sessment Study in Vitro and in Vivo. Food Res. Int..

[15] Johnson W.W., Harris T.M., Guengerich F.P. (1996). Kinetics and Mechanism of Hydrolysis of Aflatoxin B1 exo-8,9-Epoxide and Rearrangement of the Dihydrodiol. J. Am. Chem. Soc..

[16] Higgins D., Dworkin J. (2012). Recent Progress in Bacillus subtilis Sporulation. FEMS Microbiol. Rev..

[17] Guan Z.-B., Shui Y., Song C.-M., Zhang N., Cai Y.-J., Liao X.-R. (2015). Efficient Secretory Production of CotA-Laccase and Its Application in the Decolorization and Detoxification of Industrial Textile Wastewater. Environ. Sci. Pollut. Res..

[18] Välimets S., Pedetti P., Virginia L.J., Hoang M.N., Sauer M., Peterbauer C. (2023). Secretory Expression of Recombinant Small Laccase Genes in Gram-Positive Bacteria. Microb. Cell Fact..

[19] Sun F., Yu D., Zhou H., Lin H., Yan Z., Wu A. (2023). CotA Laccase from Bacillus licheniformis ZOM-1 Effectively Degrades Zearalenone, Aflatoxin B1 and Alternariol. Food Control.

[20] Sun C.-C., Dong W.-R., Shao T., Li J.-Y., Zhao J., Nie L., Xiang L.-X., Zhu G., Shao J.-Z. (2017). Peroxiredoxin 1 (Prx1) Is a Du-al-Function Enzyme by Possessing Cys-Independent Catalase-like Activity. Biochem. J..

[21] Takeda K., Nishiyama Y., Yoda K., Watanabe T., Nimura-Matsune K., Mura K., Tokue C., Katoh T., Kawasaki S., Niimura Y. (2004). Distribution of Prx-Linked Hydroperoxide Reductase Activity among Microorganisms. Biosci. Biotechnol. Biochem..

[22] Hullo M.-F., Moszer I., Danchin A., Martin-Verstraete I. (2001). CotA of Bacillus subtilis Is a Copper-Dependent Laccase. J. Bacteriol..

[23] Wang Y., Zhang Q., Pei J., Su Y., Adegoke T.V., Wang Y. (2025). Rational Screening of Four Peroxidases with High Aflatoxin B1 Degradation Efficiency via Integrated Computational Simulations. J. Agric. Food Chem..

[24] Ma R., Peng W., Xie Y., Muzaffar N., Ma W., Yang Y., Li Q., Jia H. (2025). Mechanistic Insights into Aflatoxin B1 and M1 Degradation by Bacillus subtilis HNGD-Mq02 from Wheat Koji and Its Application in Food Detoxification. J. Agric. Food Chem..

[25] Zhao W., Wu Y., Wang H., Yan Z. (2025). Degradation of Aflatoxin B1 by Recombinant Laccase AnLI from Aspergillus Niger SF951 Expressed in Escherichia coli BL21: A Mechanism Assessment in Silico and in Vitro. Food Biosci..

[26] Loi M., Fanelli F., Cimmarusti M.T., Mirabelli V., Haidukowski M., Logrieco A.F., Caliandro R., Mule G. (2018). In Vitro Single and Combined Mycotoxins Degradation by Ery4 Laccase from Pleurotus Eryngii and Redox Mediators. Food Control.

[27] Zhao L.H., Guan S., Gao X., Ma Q.G., Lei Y.P., Bai X.M., Ji C. (2011). Preparation, Purification and Characteristics of an Aflatoxin Degradation Enzyme from Myxococcus Fulvus ANSM068: An Aflatoxin Degradation Enzyme. J. Appl. Microbiol..

[28] Wang X., Bai Y., Huang H., Tu T., Wang Y., Wang Y., Luo H., Yao B., Su X. (2019). Degradation of Aflatoxin B1 and Zearalenone by Bacterial and Fungal Laccases in Presence of Structurally Defined Chemicals and Complex Natural Mediators. Toxins.

[29] Xu X., Lin P., Lu Y., Jia R. (2025). Degradation and Detoxification of Aflatoxin B1 by Two Peroxidase Enzymes from Irpex Lacteus F17. Bioprocess Biosyst. Eng..

[30] Wang J., Ogata M., Hirai H., Kawagishi H. (2011). Detoxification of Aflatoxin B1 by Manganese Peroxidase from the White-Rot Fungus Phanerochaete Sordida YK-624: Detoxification of AFB1 by MnP. FEMS Microbiol. Lett..

[31] Qin X., Su X., Tu T., Zhang J., Wang X., Wang Y., Wang Y., Bai Y., Yao B., Luo H. (2021). Enzymatic Degradation of Multiple Major Mycotoxins by Dye-Decolorizing Peroxidase from Bacillus subtilis. Toxins.

[32] Yehia R.S. (2014). Aflatoxin Detoxification by Manganese Peroxidase Purified from Pleurotus Ostreatus. Braz. J. Microbiol..

[33] You C., Zhang X.-Z., Zhang Y.-H.P. (2012). Simple Cloning via Direct Transformation of PCR Product (DNA Multimer) to Escherichia coli and Bacillus subtilis. Appl. Environ. Microbiol..

[34] Zhang X.-Z., You C., Zhang Y.-H.P., Sun L., Shou W. (2014). Transformation of Bacillus Subtilis. Engineering and Analyzing Multicellular Systems.

[35] Crowe J., Steude Masone B., Ribbe J., Harwood C.R. (1997). One-Step Purification of Recombinant Proteins with the 6xHis Tag and Ni-NTA Resin. Basic DNA and RNA Protocols.

[36] Zhang K., Su L., Duan X., Liu L., Wu J. (2017). High-Level Extracellular Protein Production in Bacillus subtilis Using an Optimized Dual-Promoter Expression System. Microb. Cell Fact..

[37] Elemosho R., Suwanto A., Thenawidjaja M. (2021). Extracellular Expression in *Bacillus subtilis* of a Thermostable *Geobacillus stearothermophilus* Lipase. Electron. J. Biotechnol..

[38] Smith P.K., Krohn R.I., Hermanson G.T., Mallia A.K., Gartner F.H., Provenzano M.D., Fujimoto E.K., Goeke N.M., Olson B.J., Klenk D.C. (1985). Measurement of Protein Using Bicinchoninic Acid. Anal. Biochem..

[39] Bradford M.M. (1976). A Rapid and Sensitive Method for the Quantitation of Microgram Quantities of Protein Utilizing the Principle of Protein-Dye Binding. Anal. Biochem..

[40] Wingfield P.T. (2015). Overview of the Purification of Recombinant Proteins. CP Protein Sci..

[41] Afsah-Hejri L., Jinap S., Arzandeh S., Mirhosseini H. (2011). Optimization of HPLC Conditions for Quantitative Analysis of Aflatoxins in Contaminated Peanut. Food Control.

[42] Waheeb M.S., Elkhatib W.F., Yassien M.A., Hassouna N.A. (2024). Optimized Production and Characterization of a Thermostable Cellulase from Streptomyces Thermodiastaticus Strain. AMB Express.

[43] Sirin Y., Akatin M.Y., Colak A., Saglam Ertunga N. (2017). Dephytinization of Food Stuffs by Phytase of *Geobacillus* sp. TF16 Im-mobilized in Chitosan and Calcium-Alginate. Int. J. Food Prop..

[44] Ewert J., Glück C., Strasdeit H., Fischer L., Stressler T. (2018). Influence of the Metal Ion on the Enzyme Activity and Kinetics of PepA from Lactobacillus Delbrueckii. Enzym. Microb. Technol..

[45] Lv Y., Liang Q., Li Y., Li X., Liu X., Zhang D., Li J. (2021). Effects of Metal Ions on Activity and Structure of Phenoloxidase in Penaeus Vannamei. Int. J. Biol. Macromol..

[46] Loi M., De Leonardis S., Ciasca B., Paciolla C., Mulè G., Haidukowski M. (2023). Aflatoxin B1 Degradation by Ery4 Laccase: From In Vitro to Contaminated Corn. Toxins.

[47] Kim S., Chen J., Cheng T., Gindulyte A., He J., He S., Li Q., Shoemaker B.A., Thiessen P.A., Yu B. (2021). PubChem in 2021: New Data Content and Improved Web Interfaces. Nucleic Acids Res..

[48] Waterhouse A., Bertoni M., Bienert S., Studer G., Tauriello G., Gumienny R., Heer F.T., de Beer T.A.P., Rempfer C., Bordoli L. (2018). SWISS-MODEL: Homology Modelling of Protein Structures and Complexes. Nucleic Acids Res..

[49] Rosignoli S., Paiardini A. (2022). Boosting the Full Potential of PyMOL with Structural Biology Plugins. Biomolecules.

[50] Trott O., Olson A.J. (2010). AutoDock Vina: Improving the Speed and Accuracy of Docking with a New Scoring Function, Efficient Optimization, and Multithreading. J. Comput. Chem..

