# Supplementary material for: Thermostable Oxidoreductases CotA and Prx Enable Synergistic and Peroxide-Enhanced Degradation of Aflatoxin B1
Source: Toxins (Basel). 2026 Apr 22;18(5):193. doi: 10.3390/toxins18050193 (PMC13211459; doi:10.3390/toxins18050193)
Supplement: Supplementary file 1 [file toxins-18-00193-s001.zip › toxins-4238324-supplementary.pdf]

Supplementary materials: Thermostable Oxidoreductases CotA and Prx Enable Synergistic and Peroxide-Enhanced Degradation of Aflatoxin B1

Xinyue Zhang [1,†], Yufan Yang [1,†], Yongping Jiang [1], Lingfang Shi [1], Haolan Du [1], Antonio Francesco Logrieco [2], Antonio Moretti [2], Susu Han [1,*] and Fuguo Xing [1,*]

[1] Key Laboratory of Agro-products Quality and Safety Control in Storage and Transport Process, Ministry of Agriculture and Rural Affairs, Institute of Food Science and Technology, Chinese Academy of Agricultural Sciences, Beijing 100193, China; zxyyue1213@163.com (X.Z.); dddkayyf@163.com (Y.Y.); 13709651670@163.com (Y.J.); 19832297939@163.com (L.S.); duhaolan@caas.cn (H.D.); hansusu@caas.cn (S.H.);
[2] Xianghu Laboratory, Zhejiang Provincial Laboratory of Agriculture, Hangzhou 311231, China; antoniofrancesco.logrieco@cnr.it (A.L.); antoniomoretti@cnr.it (A.M.)
* Correspondence: xingfuguo@caas.cn; Tel.: +86-10-62811868
† These authors contributed equally to this work.

Figure S1

Received: date
Revised: date
Accepted: date
Published: date

Citation: To be added by editorial staff during production.

Copyright: © 2025 by the authors. Submitted for possible open access publication under the terms and conditions of the Creative Commons Attribution (CC BY) license (https://creativecommons.org/licenses/by/4.0/).

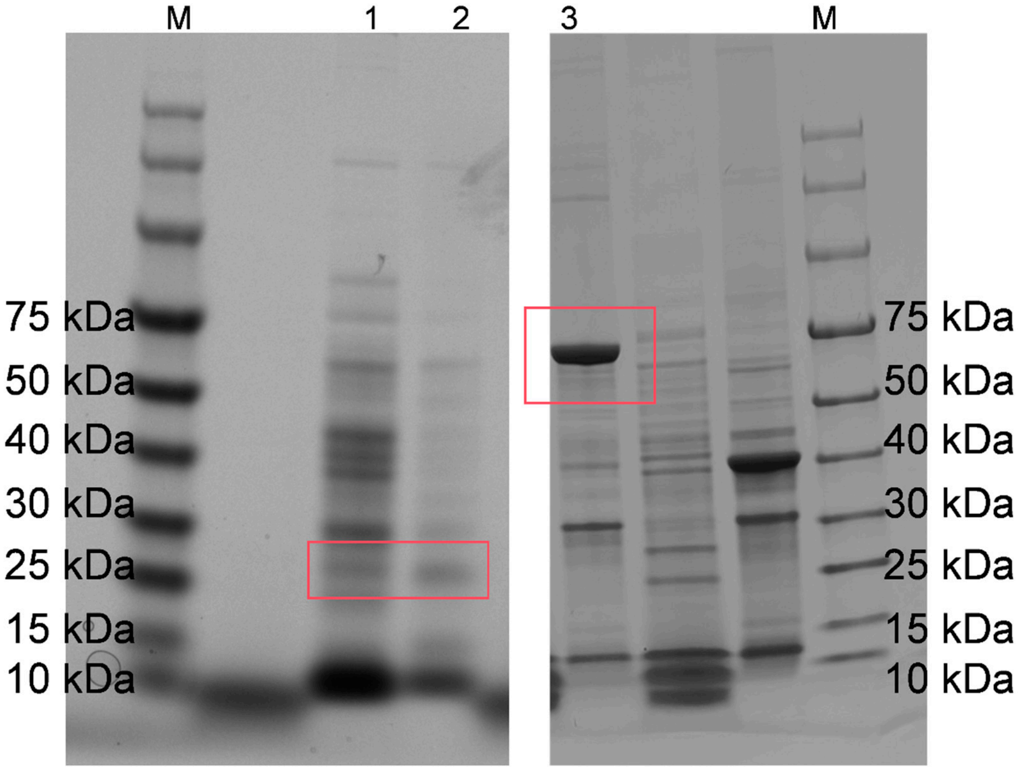

Figure S1. SDS-PAGE of CotA and Prx by B.sublitis sck6. M, marker; lane 1: culture of sck6 (pMA5-Prx); lane 2: culture supernatant of sck6 (pMA5-Prx); lane 3: culture supernatant of sck6 (pMA5-CotA).

Figure S2

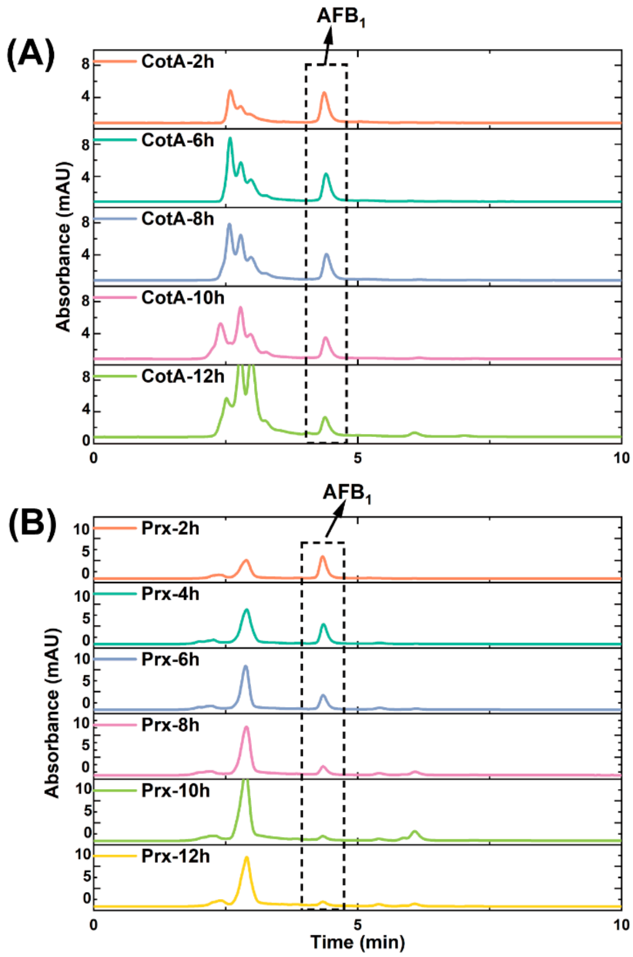

Figure S2. HPLC chromatograms of the time course of AFB_1 degradation by CotA (A) and Prx (B).

Table S1. Docking score and hydrogen bonding distances between enzymes and AFB1.

Enzyme–mycotoxin	Docking score (kcal/mol)	amino acid residue	H-bond length (Å)
CotA-AFB1	-8.20	Glu-179	2.3
		Lys-178	3.5
		Lys-178	2.0
		Arg-64	2.6
Prx-AFB1	-7.39	Arg-136	2.7
		Lys-139	2.0
